# The assistance gaps in combating COVID-19 in Brazil: for whom, where and when vaccination occurs

**DOI:** 10.1186/s12879-022-07449-5

**Published:** 2022-05-17

**Authors:** Rafael da Silveira Moreira, Emilly Guaris Costa, Lucas Fernando Rodrigues dos Santos, Luiz Henrique Lélis Miranda, Raiany Rodrigues de Oliveira, Ricardo Fusano Romão, Rodolfo Ferreira Cozer, Sarah Cavalcanti Guedes

**Affiliations:** 1 Public Health Department, Instituto Aggeu Magalhães, Fundação Oswaldo Cruz, Recife, Pernambuco, Brazil; 2 Centre for Medical Sciences, Universidade Federal de Pernambuco, Recife, Pernambuco, Brazil

**Keywords:** COVID-19 pandemics, Immunisation programs, Vaccination coverage, Spatial analysis, Social vulnerability, Brazil

## Abstract

**Background:**

Following the emergence of the COVID-19 pandemic, the number of infected Brazilian people has increased dramatically since February 2020, with Brazil being amongst the countries with the highest number of cases and deaths. Brazilian vaccination began in January 2021, aimed at priority groups. This study analysed the spatial and temporal evolution of vaccination in Brazil between the 3rd and 21st epidemiological weeks (EW) of 2021.

**Methods:**

Spatial and temporal analyses were performed comprising 19 EW. Cases were structured into priority groups—elderly population (EP); healthcare workers (HW); indigenous and quilombola populations (I/Q), dose, vaccine (CoronaVac or AstraZeneca), and place of vaccination. A sweep test was performed to identify vaccination rate clusters. Vaccination rates (VR) were calculated according to a spatial window for each Health Region, indicating clusters above/below expected VR. Based on the discrete Poisson probability model, spatial analysis was performed to detect high/low VR clusters, which were converted into Kernel maps. Points were generated from SaTScan analyses associated with Health Region centroids. Temporal analysis of VR was carried out to identify significant trends, and results were converted into temporal cluster graphs. P-value ≤ 0.05 was adopted.

**Results:**

Southeast region concentrated most of the vaccines of EP and HW, followed by the Northeast. The latter region had the largest contingent of I/Q vaccinated. In all priority age groups and all regions, a higher percentage of complete CoronaVac vaccination schedules were observed compared with AstraZeneca. The temporal analysis identified high VR clusters of CoronaVac first and second dose in the early weeks, except for the EP; of AstraZeneca first dose, only for HW in the early weeks, and for EP and I/Q in the final weeks; of AstraZeneca second dose for all priority groups in the final weeks. I/Q populations had the lowest general VR. The spatial profile of VR indicated significant regional cluster differences between the priority groups.

**Conclusion:**

This study highlights the importance of establishing vaccination priority groups, considering the asymmetries that a pandemic can trigger, notably in vast geographic areas, to contemplate the main objective of vaccination: to prevent casualties.

## Background

The novel coronavirus emerged on 29 December 2019, with the report of four cases of severe acute respiratory syndrome (SARS) of unknown aetiology in Wuhan city, Hubei province, China. On 31 January 2020, there were 213 deaths reported globally [1]. In Brazil, the first case of COVID-19 was reported by the Ministry of Health (MH) on 25 February 2020, with the number of people infected increasing dramatically since then [2]. By the end of June 2020, Brazil ranked second globally for both the number of confirmed cases and the number of deaths, only behind the United States [3].

Since the beginning of the pandemic, scientists have joined forces to develop a safe and effective vaccine. This effort allowed the start of research and testing in an unprecedented time, resulting in some vaccines being approved for use as early as 2021 [4]. In Brazil, the first authorised vaccine was CoronaVac, produced by the private Chinese company Sinovac in partnership with the Butantan Institute (São Paulo, Brazil), with 50.4% efficacy. The CoronaVac is based on inactivated coronaviruses that can no longer replicate, but their spike protein—used to enter human cells—remains intact. It works by teaching the immune system to make antibodies against the severe acute respiratory syndrome coronavirus 2 (SARS-CoV-2). With an application performed in two doses, with an interval of 28 days, CoronaVac had wide distribution in Brazil, especially among healthcare workers [4].

Another critical element for large-scale vaccination in Brazil was the vaccine produced by the British company AstraZeneca and designed by the University of Oxford. The Vaxzevria, also known as Covishield, is a non-replicating chimpanzee adenovirus-vectored vaccine based on the coronavirus's genetic instructions for building the spike protein. The instructions are stored in double-stranded DNA, which is added to a modified version of adenovirus. It has an efficacy of 76% after the two doses of application, 12 weeks apart. It received Brazilian approval on 13 March and began to assume gaps in the country's vaccination, which are still incipient [5].

Vaccination began in Brazil on 19 January 2021 [6]. On 03 April 2022, the total number of Brazilians who received at least one dose of the vaccine Against COVID-19 was 181.3 million, with 76% of the Brazilian population having received two doses of the vaccine [7]. Considering people with a complete initial protocol and people only partially vaccinated, approximately 65% are between 20 and 59 years old; 0.56% are under five years old, 18% are between 5 and 19 years old, and 15% are over 60 years old [8].

Considering the urgency for broad vaccination coverage in a pandemic context while insufficient supply and production, the creation of priority groups were necessary [9, 10]. Based on World Health Organization (WHO) guidelines [9], social workers and healthcare workers were established as a priority group, which in Brazil represents a workforce equivalent to more than 3.5 million people [11]. The vaccination of this group is of utmost importance since they are on the front line of fighting the virus, and the greater exposure is directly linked to greater chances of contamination and mortality by COVID-19. Data from the WHO and the Brazilian MH indicated that 307,000 Brazilian healthcare professionals were infected by the coronavirus [12].

The second group corresponds to the elderly or people with comorbidities, depending on the social risk. According to the Brazilian Institute of Geography and Statistics (IBGE), about 7% of the population is older than 60 years. In this perspective, vaccination was initiated in a staggered method, respecting each state's age range and distribution [13].

Due to their susceptibility to the virus and socially vulnerable status, traditional indigenous and *quilombola* populations were also defined as priority groups for vaccination in Brazil. The 2010 national census estimated approximately 897 thousand indigenous people in Brazil and 5972 quilombola communities [13]. An analysis published by the end of 2020 recorded 29,008 cases and 532 deaths of indigenous people in Brazil from COVID-19 [14].

The way the severe health situation was managed in several countries around the world and the population's behaviour in the face of the guidelines given by national and global public health agencies also had an enormous impact. Research conducted by the Lowy Institute, Australia, analysed the responses of 98 countries to COVID-19 through data compiled in the nine months after confirmation of the 100th case of the disease. In January 2021, Brazil received a score of 4.3 compared to 98 countries in the performance of pandemic management, while the country with the highest score, New Zealand, received 94.4 points [15].

According to Boschiero et al*.* [16], in a recent review of the political and social panorama of the pandemic in Brazil, the management of the dissemination of COVID-19 failed and the main points related to the chaotic situation in the country were: poor management of the public health system, disparities between public and private health infrastructure, lack of mass testing, lack of preparation and planning to implement isolation measures, political instability, deterioration of the Ministry of Health and bad attitudes of the federal government, such as the adoption of anti-scientific actions, minimisation of the severity of COVID-19 and promotion of proven ineffective drugs for the treatment of the disease.

In the opposite direction, the population-based epidemiological enquiries presented themselves as an essential strategy for tracking coronavirus infection and assisting mechanisms involved in the severity criteria and hospital admission. From the development of these surveys, it was possible to readjust the control measures, outline goals of non-pharmacological care and better target resources [17–19]. Thus, investigating the pace of vaccination against COVID-19, overlapping demographic characteristics to epidemiological criteria, is essential to understand the effectiveness of vaccination, its progress, and the gaps in this process [20]. Therefore, this study aimed to analyse the spatial and temporal evolution of vaccination in Brazil between the 3rd and 21st epidemiological weeks of 2021.

## Methods

An ecological study was conducted with an analysis of spatial and temporal groupings. The units of analysis were the Health Regions (HR) of Brazil, characterised as continuous geographical space formed by the union of bordering municipalities according to cultural, economic, and social identities. In addition, communication networks and shared transport infrastructure are considered to integrate the organisation, planning and execution of health actions and services [21]. Thus, the 449 HR of Brazil were considered for this work. The study period was from 17 January 2021 to 29 May 2021, comprising 19 weeks, from the third to the 21st week, based on the 2021 epidemiological calendar of the Brazilian Information System of Notifiable Diseases—SINAN (http://portalsinan.saude.gov.br/). This time frame was selected considering the first vaccination dose against COVID-19 in Brazil and the date when the statistical data treatment started.

Data on cases categorised by residence were collected from the Brazilian public health system (SUS) department of informatics website using the OpenDATASUS repository (https://opendatasus.saude.gov.br). Those were structured according to priority groups, epidemiological week of vaccination, dose, the vaccine used—CoronaVac or AstraZeneca, age groups, and place of vaccination. In addition, demographic data of priority groups (2020) stratified by population aged 60 and older (the elderly) and by health workers were collected through the SUS department of informatics OpenDataSUS repository (http://www.datasus.gov.br). The demographic data of the indigenous and quilombola communities (2019) were collected from the information database on Indigenous Peoples and Quilombolas of the IBGE repositories (https://www.ibge.gov.br).

The choice of priority groups analysed here followed the strategic planning of the Brazilian Ministry of Health used to confront the pandemic of COVID-19, which led to these groups making up the vast majority of those immunised during the period studied. Moreover, it aimed to bring more social inclusion and diversity to this work by analysing the vaccination profile among indigenous people and quilombolas, population clusters traditionally neglected in the literature, and social and health care matters.

The vaccination rates per 100 inhabitants and their temporal and spatial distribution were calculated for all the priority groups. For the spatial distribution, coordinates corresponding to centroids of each HR were collected in the form of decimal measures of latitude and longitude.

The analyses were conducted according to the performance of the two vaccines, CoronaVac and AstraZeneca, arranged in two comparative blocks. The same variable was studied for each block: vaccination performance according to the priority groups for both doses. Although the two immunisers evaluated were not the only ones available in Brazil during the study period, they accounted for almost all applications. At the same time, vaccines from other manufacturers were limited to small population groups still in the early stages of clinical trials and, therefore, not widely available.

The sweep test was chosen to evaluate the spacial arrangement by identifying risk clusters. Thus, vaccination rates were recalculated for the construction of expected rates considering the exposed population and its geographical neighbourhood matrix, according to a certain radius of spatial scanning (spatial window). Once the expected rates were obtained, the ratio between the observed (actual) rate and the expected rate was calculated for each Health Region. Values above 1 indicate clusters with an above-expected vaccination rate, while values below 1 indicate clusters where observed rates are below expected.

The software SaTScan 9.6 was used for the analysis, based on the discrete Poisson probability model [22], to detect clusters of high and low risk of vaccination. Traditionally, SaTScan detects clusters of spatial risk or protection. However, because the analysed event is a vaccine, we replaced the term risk with rate while preserving the same interpretation of risk since the event is a favourable health outcome. The collected data were aggregated according to geographic distribution associated with HR centroid coordinates and temporal distribution associated with each epidemiological week of interest for both vaccines, loaded into the software separately.

The analyses were done by crossing the dose and priority groups variables to detect high and low clusters separately.

For the spatial analyses, a circular spatial window with 50% of the exposed population at risk was used at the beginning. After the first evaluation, this circular spatial window was corrected, and the software was loaded again with the adjusted sweep percentage, as indicated by the ideal Gini coefficient. Monte Carlo simulations (999 permutations) were considered to obtain the significance of the tests (p-value) [22]. A temporal disposition analysis of vaccination rates was performed with the same software to identify significant temporal trends. The significance level adopted was 5%.

The numerical results of temporal analyses were converted into temporal cluster graphs with clustering units adjusted for one epidemiological week. The graphic results of the spatial analyses were used to construct Kernel maps for the geographical visualisation of HRs according to the distribution of immunisation coverage by the vaccine, dose, and priority groups. Kernel estimators comprise a set of non-parametric statistical tools that allow the smoothing of a geographic surface according to the density of points belonging to a given area [23]. This procedure is based on a two-dimensional reformatting that gives the location and concentration of points as a function of predetermined and adjustable distances, allowing spatial references to be extrapolated to places where there were no points. The degree of smoothing used an adaptive radius, varying according to the density of the points and quartic function. Points were generated from SaTScan analyses associated with HR centroids. The spatial representation through Kernel maps was performed with QGIS 3.18.2 software. This set of tools allows a geographical arrangement in which it is possible to observe the concentration of phenomena that indicate a spatial agglomeration. For all the graphic and cartographic representations, it was adopted p-value ≤ 0.05.

As these are publicly available information in official national databases, aggregated by Health Regions, and without the possibility of individual identification, this work did not require approval by the Research Ethics Committee, being, therefore, in compliance with the directives of Resolution 466 of 2012 of the Brazilian National Health Council.

## Results

Table 1 shows the general vaccination data in Brazil's geographic regions for the priority groups—the elderly population (EP) group, healthcare workers (HW) group, and indigenous and quilombolas populations (I/Q) group. By the end of May 2021, 51,722,625 doses of AstraZeneca or CoronaVac vaccines had been administered to these three epidemiological groups in the country. Among the total administered in the period, 38,580,849 vaccines were applied by priority age group criterion (elderly). For the country's population declared or considered indigenous and quilombolas, 1,176,173 doses were destined. Health workers received 11,965,603 doses of the two vaccines.

**Table 1:** Distribution of vaccine doses during the evaluated period by geographic region for priority groups. Brazil, 2021

**Region**	**Group**	**CoronaVac**				**AstraZeneca**				
		**1st dose n (%)**	**2nd dose n (%)**	**Fully vaccinated**	**VR**	**1st dose n (%)**	**2nd dose n (%)**	**Fully vaccinated**	**VR**	**Total**
	EP	750,200 (5.1)	650,326 (5.1)	86.69%	39.56	608,189 (6.0)	153,563 (13.9)	25.25%	9.34	2,162,258 (5.6)
	60—64	210,856	173,437	82.25%	30.96	215,295	10,185	4.73%	1.82	609,773
	65—69	195,078	166,828	85.52%	40.23	150,793	29,303	19.43%	7.06	542,002
N	70—74	141,272	123,985	87.76%	43.89	101,189	41,880	41.39%	14.83	408,326
	75—79	95,142	86,415	90.83%	46.01	65,160	30,020	46.07%	15.98	276,737
	80 +	107,852	99,661	92.40%	50.11	75,732	42,175	55.69%	21.2	325,420
	HW	264,925 (6.4)	234,436 (6.3)	88.49%	58.61	133,992 (4.4)	61,198 (5.7)	45.67%	15.3	694,551 (5.8)
	I/Q	135,357 (39.3)	90,021 (33.4)	66.51%	26.27	168,625 (29.9)	2,280 (12.6)	0.38%	0.18	394,638 (33.6)
	EP	3,734,105 (25.4)	3,187,557 (25.1)	85.36%	43.97	2,343,992 (23.3)	240,741 (21.8)	10.31%	3.32	9,506,395 (24.6)
	60—64	491,638	372,465	75.76%	17.09	1,095,861	6,657	0.61%	0.3	1,996,621
	65—69	1,084,657	904,680	83.40%	52.75	504,901	3,778	0.75%	0.22	2,498,016
NE	70—74	1,001,896	869,803	86.82%	65.32	125,146	3,317	2.65%	0.25	2,000,162
	75—79	640,426	571,529	89.24%	62.53	158,042	32,892	20.81%	3.6	1,402,889
	80 +	515,488	469,080	91.00%	42.24	460,042	194,097	42.19%	17.48	1,638,707
	HW	1,123,282 (27.2)	980,223 (26.4)	87.26%	59.01	680,190 (22.3)	210,086 (19.6)	30.88%	11.69	2,993,781 (25.0)
	I/Q	100,211 (29.1)	84,415 (31.3)	84.23%	36.87	291,254 (51.7)	1,217 (6.7)	0.42%	0.53	477,097 (40.6)
	EP	6,827,568 (46.4)	5,813,281 (45.7)	85.14%	40.65	4,673,492 (46.4)	476,824 (43.3)	10.20%	3.33	17,791,165 (46.1)
	60—64	418,011	288,525	69.02%	6.51	2,563,521	16,045	0.62%	0.36	3,286,102
	65—69	2,293,383	1,831,421	79.86%	52.52	1,087,506	14,332	1.32%	0.41	5,226,642
SE	70—74	2,006,187	1,760,628	87.76%	69.29	86,999	11,163	12.83%	0.44	3,864,977
	75—79	1,103,770	1,009,949	91.50%	59.96	315,599	120,220	38.09%	7.14	2,549,338
	80 +	1,006,217	922,758	91.70%	42.77	619,867	315,064	50.83%	14.6	2,863,906
	HW	1,809,235 (43.8)	1,638,179 (44.1)	90.54%	49.64	1,504,349 (49.4)	473,990 (44.2)	68.49%	14.36	5,425,753 (45.3)
	I/Q	21,766 (6.3)	19,498 (7.2)	89.58%	19.92	67,072 (11.9)	1,039 (5.7)	1.55%	1.06	109,375 (9.3)
	EP	1,029,113 (7.0)	943,128 (7.4)	91.64%	47.6	717,314 (7.1)	95,018 (8.6)	13.25%	4.8	2,784,573 (7.2)
	60—64	107,534	84,939	79.00%	12.87	423,699	5,874	1.39%	0.89	631,046
	65—69	318,153	286,917	90.18%	57.86	123,310	3,814	3.09%	0.77	732,194
CW	70—74	296,639	280,162	94.45%	80.6	27,095	3,796	14.01%	1.09	607,692
	75—79	152,013	144,937	95.35%	63.69	60,422	29,984	49.62%	12.87	387,356
	80 +	154,774	146,173	94.44%	58.39	82,788	51,550	62.27%	20.59	435,285
	HW	314,342 (7.6)	296,655 (8.0)	86.91%	45.63	335,254 (11.0)	128,553 (12.0)	39.35%	19.78	1,074,804 (9.0)
	I/Q	58,054 (16.9)	50,220 (18.6)	86.06%	35.08	20,419 (3.6)	13,468 (74.4)	18.86%	1.79	124,448 (10.6)
	EP	2,365,842 (16.1)	2,115,986 (16.6)	89.44%	42.15	1,718,530 (17.1)	136,100 (12.3)	7.92%	2.71	6,336,458 (16.4)
	60—64	143,740	69,700	48.49%	4.49	1,051,051	3,047	0.29%	0.2	1,267,538
	65—69	900,508	747,100	82.97%	60.41	351,166	1,765	0.50%	0.14	2,000,539
S	70—74	711,984	642,083	90.18%	70.85	30,871	923	2.99%	0.1	1,385,861
	75—79	407,658	372,413	91.36%	62	71,336	16,067	22.52%	2.67	867,474
	80 +	201,952	284,690	140,97%^a^	39.31	214,106	114,298	53.38%	15.78	815,046
	HW	619,281 (15.0)	566,713 (15.2)	91.51%	51.52	391,701 (12.9)	199,019 (18.6)	50.81%	18.09	1,776,714 (14.8)
	I/Q	28,877 (8.4)	25,392 (9.4)	87.93%	32.62	16,283 (2.9)	108 (1.9%)	0.66%	0.14	70,660 (6.0)
Total EP		14,706,828 (76.7)	12,710,278 (76.1)	86.42%	42.09	10,061,497 (73.6)	1,102,246 (50.5)	10.96%	3.65	38,580,849 (74.6)
Total 60–64		1,371,779	989,066	71.88%	10.54	5,349,427	41,808	0.78%	0.44	7,749,080
Total 65–69		4,791,779	3,936,946	82.16%	53.57	2,217,676	52,992	2.39%	0.72	10,999,393
Total 70–74		4,157,978	3,676,661	88.42%	67.98	371,300	61,079	16.45%	1.13	8,267,018
Total 75–79		2,399,009	2,185,243	91.09%	60.46	670,559	229,183	34.18%	6.34	5,483,994
Total 80 +		1,986,283	1,922,362	96.78%	43.29	1,452,535	717,184	49.37%	16.15	6,078,364
Total HW		4,131,065 (21.5)	3,716,206 (22.3)	89.96%	50.9	3,045,486 (22.3)	1,072,846 (49.2)	64.77%	14.69	11,965,603 (23.1)
Total I/Q		344,265 (1.8)	269,546 (1.6)	78.30%	30.27	556,841 (4.1)	5,521 (0.3)	0.99%	0.62	1,176,173 (2.3)
Total per dose		19,182,158 (100.0)	16,696,030 (100.0)	87.04%	**–**	13,663,824 (100.0)	2,180,613 (100.0)	15.96%	**–**	51,722,625 (100.0)
Total		35,878,188 (100.0)				15,844,437 (100.0)				51,722,625 (100.0)

VR: Vaccination rate; N: North region; NE: Northeast region; SE: Southeast region; CW: Central-West region; S: South region; EP: elderly population; HW: healthcare workers; I/Q: indigenous and quilombolas populations

Dose and total data were expressed as absolute numbers with each group's regional participation percentage in parentheses. Data on incomplete vaccination status were expressed as a percentage of the comparison between doses. Data on vaccination rates were expressed in doses per 100 inhabitants, considering only individuals with complete vaccination status

^a^ Numerical inconsistency verified in the database

The Southeast (SE) region concentrated most of the vaccines for the elderly group (46.1%) and health workers (45.3%) in the country. In this region, the vaccination coverage rate for the EP was 40.65 doses/100 inhabitants for CoronaVac and 3.33 doses/100 inhabitants for AstraZeneca, considering only complete vaccination schedules in the period studied. In the elderly group, 14.86% of incomplete vaccination schedules were observed for CoronaVac. Such count was 89.90% for AstraZeneca, the second-highest observed in the country during the study, only behind the South (S) region.

The Northeast (NE) was the vice-leader in immunisations, contributing to the elderly and health workers groups with 24.6% and 25% of doses, respectively. The largest vaccinated population contingent of indigenous and quilombolas was in this region (40.6%). However, 15.76% of the schedules with CoronaVac and 99.58% for AstraZeneca were incomplete for this priority group. On the other hand, a CoronaVac vaccination coverage rate for the elderly group of 43.97 doses/100 inhabitants was observed, the highest in the country.

The highest percentages of incomplete AstraZeneca vaccination schedules were found among indigenous and quilombola populations, with values above 98% in all country's five geographic regions. A vaccination rate of 39.56 doses of CoronaVac/100 inhabitants was observed for the elderly group in the North (N) region, the lowest in Brazil. Whereas for AstraZeneca, the region reached 9.34 doses/100 inhabitants for the same priority group, the highest in the country. For the elderly group, the Central-West (CW) region obtained the highest immunisation rate for CoronaVac, with 47.6 doses/100 inhabitants. In addition, 4.80 doses/100 inhabitants were found for AstraZeneca, the second-highest in the country.

The 65–69 age group was the one that received the most doses in the analysed period, with 10,999,393 vaccines applied and with a substantial difference between complete vaccination schedules between CoronaVac (82.16%) and AstraZeneca (2.39%). On the other hand, those aged 75–79 had 5,483,994 doses applied, the lowest number among the elderly group, with 34.18% and 91.09% of complete schemes from AstraZeneca and CoronaVac, respectively.

In all priority age groups and country regions, a higher percentage of complete CoronaVac vaccination schedules was observed than AstraZeneca. The South is also the region with the lowest vaccination rate for AstraZeneca, with 2.71 doses per 100 inhabitants and only 0.2 doses for the 60–64 age group.

**Fig. 1:**
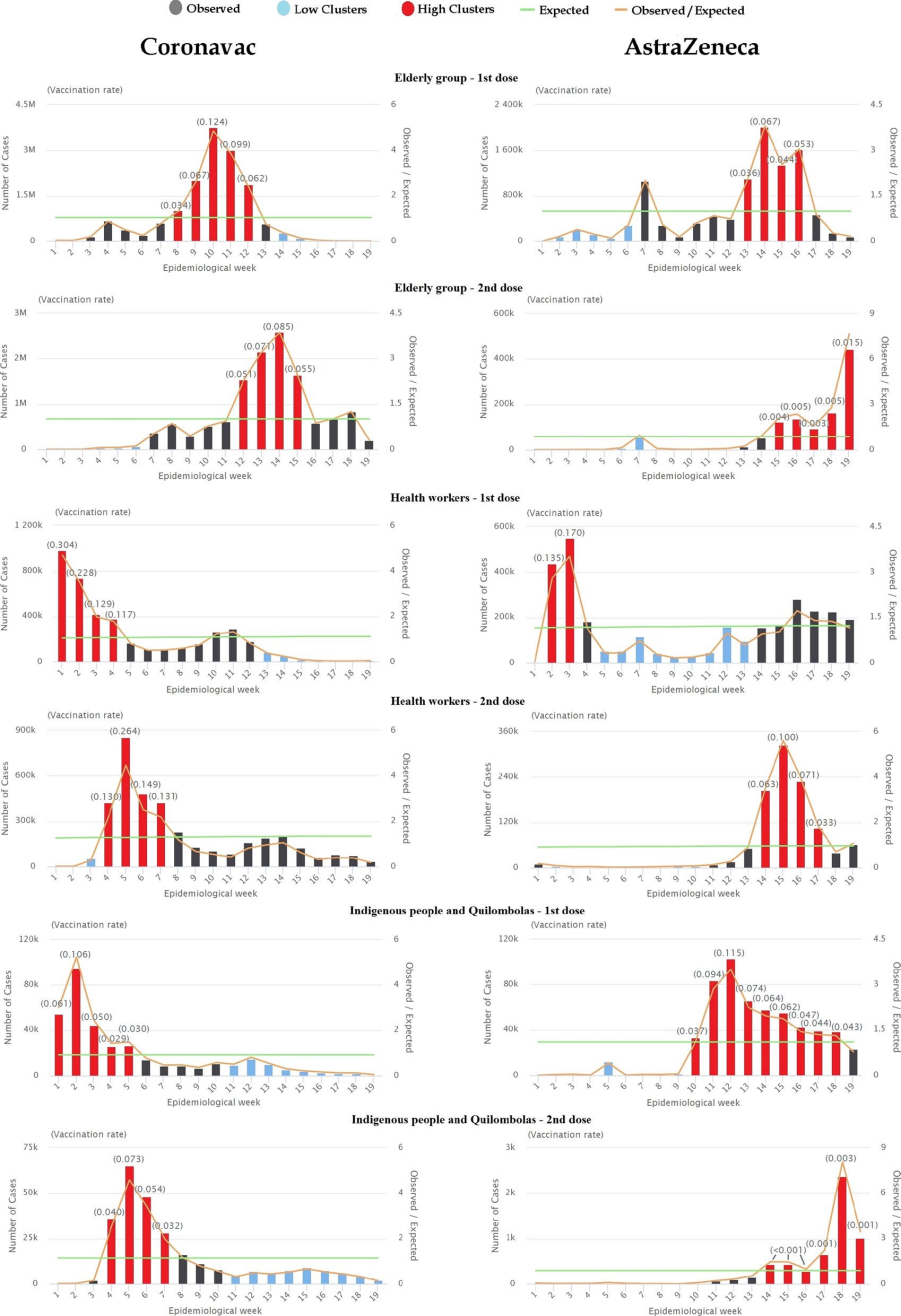
Temporal representation of the epidemiological weeks according to the clusters of vaccination rate (VR). Brazil, 2021

The temporal analysis of vaccination rates (VR) of CoronaVac and AstraZeneca doses in the groups of interest during the first five months of 2021 is shown in Fig. 1. Regarding the first dose of CoronaVac for the elderly group, one temporal cluster of high VR was detected in the country (p = 0.001) during epidemiological weeks 8–12, with a relative risk (RR) of 10.70. However, from week 14 until week 19, a low temporal cluster was observed (RR = 0.066; p = 0.001). The two other criteria analysed presented both a high incidence cluster at the beginning of the analysis – from weeks 1–4 for HW (RR = 6.08; p = 0.001) and weeks 1–5 for I/Q (RR = 7.00; p = 0.001); and a low incidence cluster from weeks 13–19 (RR = 0.19; p = 0.001) and weeks 11–19 (RR = 0.084; p = 0.001) for HW and I/Q, respectively.

In the CoronaVac second dose vaccination for the elderly group, a low vaccination rate cluster was observed between weeks 1–6 (RR = 0.026; p = 0.001), with a high cluster at weeks 12–15 (RR = 6.19; p = 0.001). The low and high clusters for health workers were at weeks 1–3 (RR = 0.085; p = 0.001) and 4–7 (RR = 5.45; p = 0.001) respectively, while those for group I/Q were at 4–7 (RR = 7.19; p = 0.001) and 11–19 (RR = 0.28; p = 0.001).

Examining the first dose of AstraZeneca temporally, the healthcare workers group had a high incidence cluster in weeks 2–3 (RR = 4.21; p = 0.001) and a low one during weeks 5–13 (RR = 0.29; p = 0.001). The elderly group, on the other hand, had a low cluster during the beginning of the analysis at weeks 1–6 (RR = 0.17; p = 0.001) and a high one at weeks 13–16 (RR = 5.70; p = 0.001). The I/Q group followed the same pattern as the elderly.

**Fig. 2:**
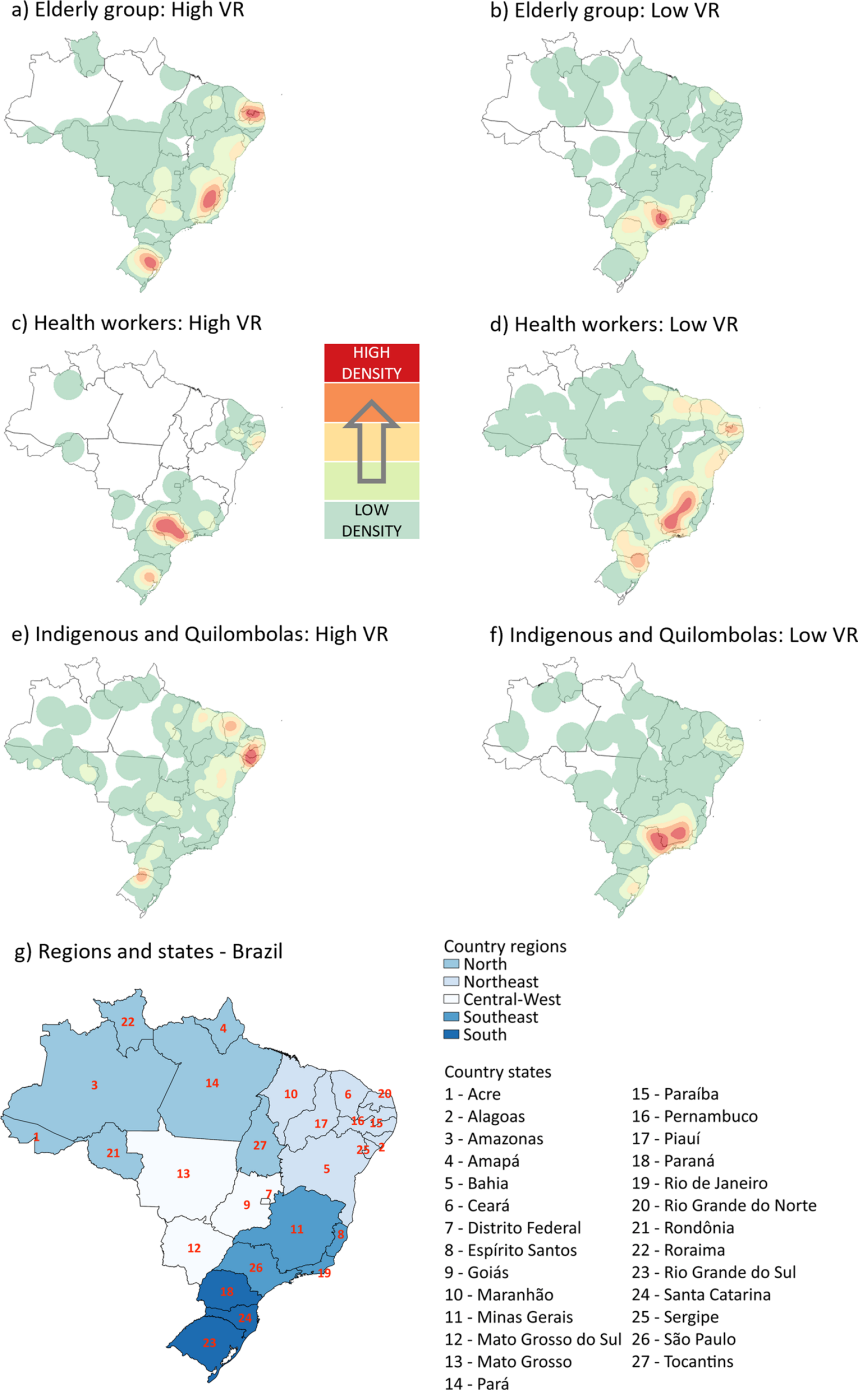
Kernel density maps with the spatial clusters of vaccination rates for the first dose of CoronaVac. Brazil, 2021

Figure 2 shows the spatial profile of vaccination rates (VR) of the first dose of CoronaVac in Brazil for the three priority groups of interest in the first five months of 2021. For the elderly group, 25 spatial clusters of high VR were detected in the country (p < 0.001). Highlights were the states of Ceará, Rio Grande do Norte and Paraíba (RR = 1.24), the eastern part of Minas Gerais (RR = 1.18), and the northeastern portion of Rio Grande do Sul (RR = 1.16), which presented the highest heat density in the Kernel representation model. On the other hand, there were 42 spatial clusters of low VR in the country (p < 0.001). Among these, the border between Minas Gerais, São Paulo and Rio de Janeiro concentrated the most significant number of low clusters (RR = 0.74 in the densest region and 0.90 in the least dense).

**Fig. 3:**
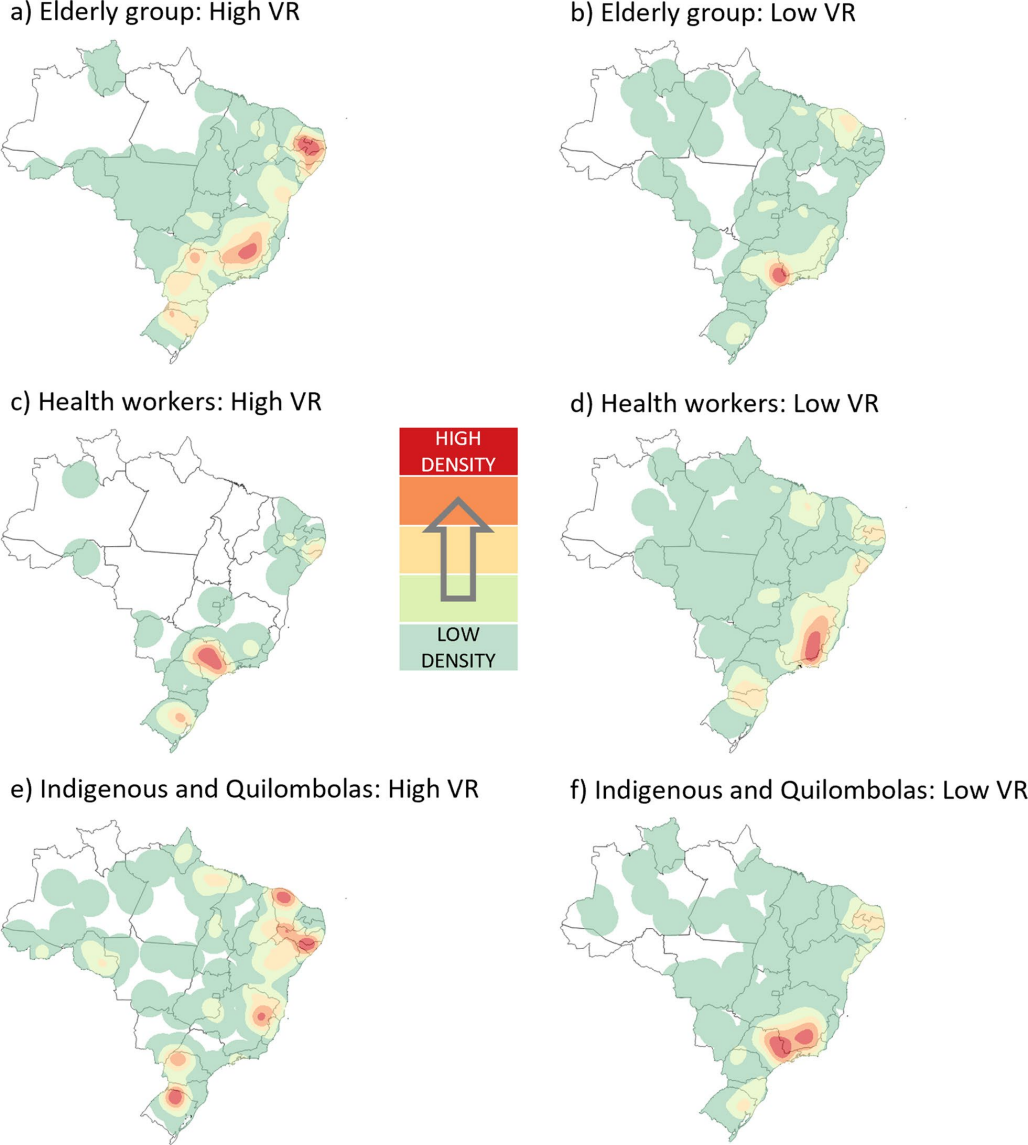
Kernel density maps with the spatial clusters of vaccination rates for the second dose of CoronaVac. Brazil, 2021

Figure 3 shows the spatial behaviour of VR by the second dose of CoronaVac in Brazil for the same groups and period as above. For the elderly group, 71 clusters of high VR were detected in the country (p < 0.04). The region formed by the interior of Rio Grande do Norte, Paraíba and Pernambuco (RR = 1.34) and the interior of Minas Gerais (RR = 1.18) remained as geographical highlights for the concentration of VR in the country. However, the South region did not maintain the density of high VR clusters for the second dose of CoronaVac in the elderly group. In contrast, a trend of accompanying geographical patterns across the country was observed in the spatial profile of low VR for the same dose, vaccine, and group. For instance, the Metropolitan Region of São Paulo (RR = 0.82) remained the most highlighted by Kernel representation among the 73 clusters identified in this series (p < 0.04).

Also, in Fig. 3, there were 21 temporal clusters of high VR (p < 0.001) for the second dose of CoronaVac in the group of health workers. It was a spatial behaviour very similar to that of the first dose, with a slight reduction in the density zone found in São Paulo, but which remains the zone with the highest concentration of these clusters (RR = 1.27). When comparing the geographic profile of low VR among doses for this same priority group, the behaviour is similar to that found in the first dose, maintaining Minas Gerais as the state with the most points (RR = 0.79). For indigenous people and quilombolas, northern Ceará (RR = 3.91), eastern Minas Gerais (RR = 1.91), and the northern portion of Rio Grande do Sul (RR = 1.36) intensified as regions of high VR for the second dose of CoronaVac in the country. At the same time, Pernambuco, Alagoas and Sergipe remain with high densities of vaccination rates (RR = 1.59).

**Fig. 4:**
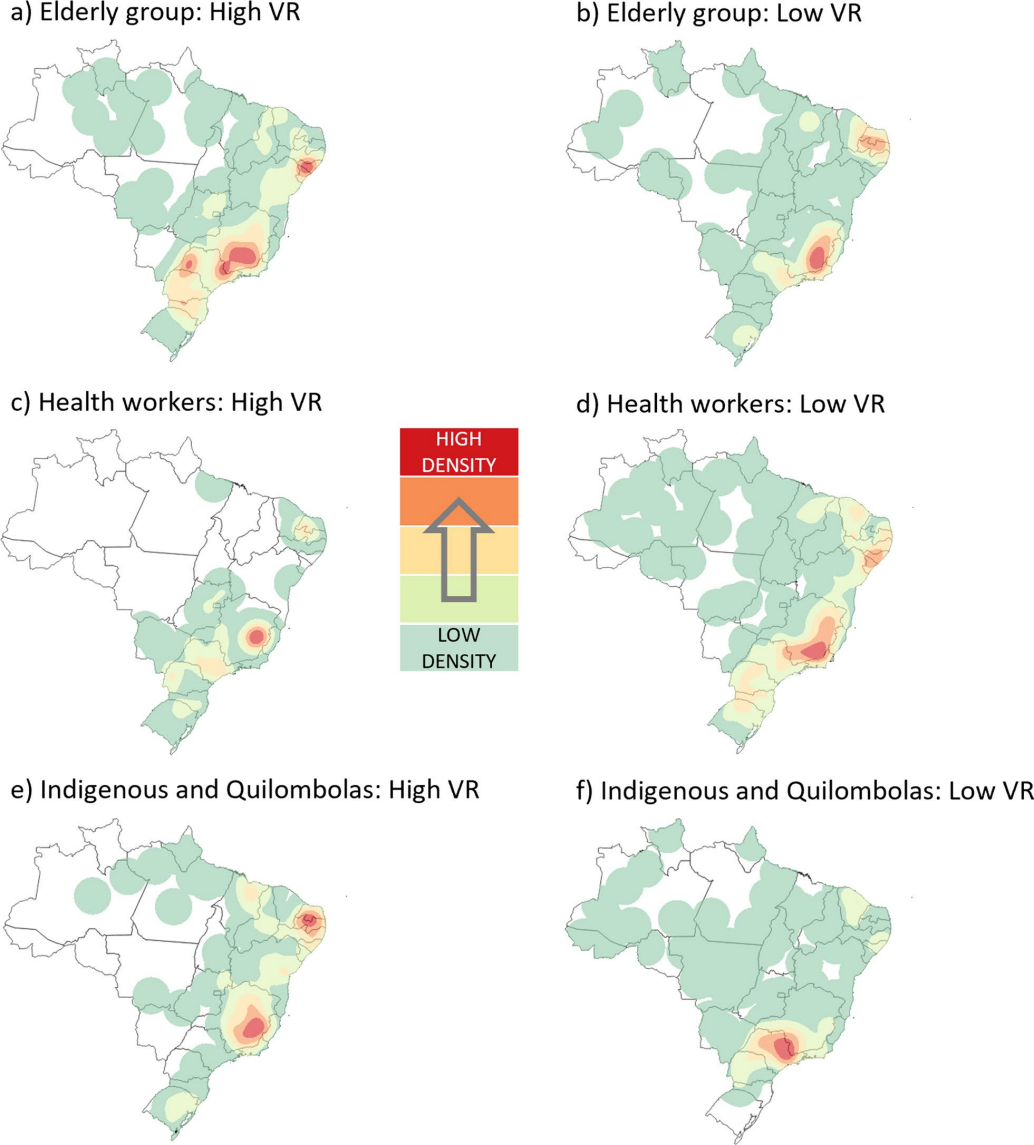
Kernel density maps with the spatial clusters of vaccination rates for the first dose of AstraZeneca. Brazil, 2021

The Kernel maps of the Health Regions according to the spatial clusters of vaccination rates of the first dose of AstraZeneca against COVID-19 for the three groups analysed in the study period are in Fig. 4. For the elderly group, it was observed that the countryside of Pernambuco, Alagoas, and Sergipe (RR = 1.24), the eastern border between Minas Gerais and São Paulo (RR = 1.21), as well as the western portion of the latter (RR = 1.23) had the highest concentration of high VR among the 51 clusters (p < 0.001) found. On the other hand, the southern zone of Minas Gerais (RR = 0.88) and, to a lesser extent, the state of Paraíba, together with Rio Grande do Norte (RR = 0.82), contained the Health Regions with the lowest VR among the 46 clusters identified (p < 0.01).

When analysing the health workers group, there were 34 clusters (p < 0.01) of high VR for the first dose of AstraZeneca, with emphasis on the eastern region of the state of Minas Gerais (RR = 1.11). On the other hand, among the 46 clusters (p < 0.001) of low VR for this same population, the southern Minas Gerais, northern Rio de Janeiro (RR = 0.63) and the region formed by the interior of Pernambuco, Alagoas and Sergipe (RR = 0.34) were highlighted. For the indigenous and quilombola group, Paraíba, Rio Grande do Norte (RR = 7.56), and eastern Minas Gerais (RR = 18.24) were the areas with the highest concentration of high VR clusters. At the same time, São Paulo and Minas Gerais (RR = 0.074) presented the highest concentration of low VR clusters in the country for this group.

**Fig. 5:**
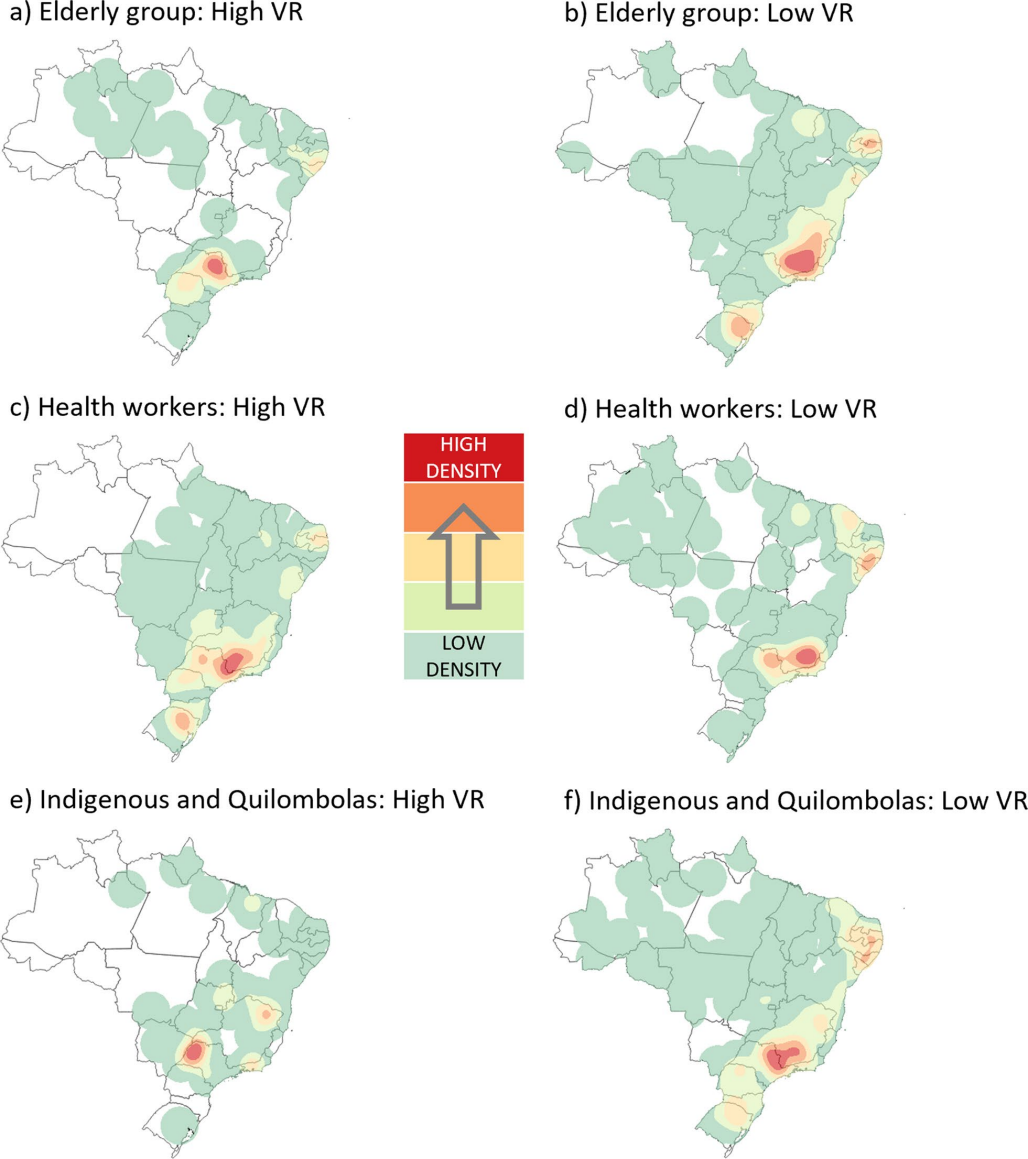
Kernel density maps with the spatial clusters of vaccination rates for the second dose of AstraZeneca. Brazil, 2021

Figure 5 demonstrates the spatial behaviour of vaccination rates with the second dose of AstraZeneca in Brazil for the three priority groups of interest until May 2021. When analysing the vaccinated population by the elderly priority group, only in São Paulo did the second dose of AstraZeneca show a set of high VR points (RR = 1.38) among the 23 clusters identified (p < 0.03). This geographic profile goes against that verified in the previous application for the same priority group, which was distributed differently in this state and still present in Minas Gerais and portions of the country's Northeast region. Among the 30 clusters of low VR (p < 0.001), the spatial distribution was more uniform when comparing doses. The southern portion of Minas Gerais (RR = 0.22) and the border between Rio Grande do Norte and Paraíba (RR = 0.39) still stood out in the Kernel representation, despite the slight increase in the density of low VR points observed in Rio Grande do Sul (RR = 0.48).

For the health workers group, an overall increase in the density of high VR clusters was observed for the second dose of AstraZeneca compared to its first, notably in the Northeast and Central-West regions of the country. In addition, there was a coalescence of high VR zones between São Paulo and Minas Gerais (RR = 1.17). When analysing the clusters of low VR, the spatial behaviour of the vaccination of health workers tends to uniformity (p < 0.03), with Minas Gerais standing out (RR = 0.71) in the concentration of low VR points. When analysing the indigenous peoples and quilombolas, it was observed that eastern São Paulo (RR = 4.03) started to concentrate the highest points of high VR for the second dose, differing vastly from the prominence observed in Minas Gerais and the northern portion of the Northeast in the first dose. For the I/Q group, 12 clusters of low LR were observed in the country (p < 0.01), with emphasis on São Paulo and Minas Gerais (RR = 0.13) and most of the Northeast region (RR = 0.12–0.13).

## Discussion

The spacial and temporal profiling of the vaccination for COVID-19 in Brazil is key to understanding who, where, and when immunisation is occurring in the country. Vaccination in Brazil presents a distinguished historical scenario, with its National Vaccination Programme (PNI) being an example for the world [24]. However, the pandemic of COVID-19 brought numerous challenges. The setback imposed by the pandemic is evident in the drop in life expectancy of the population by approximately 2 years, going to 74.8, putting Brazil back to the numbers reached in 2013 [25].

One of the criteria for defining the elderly population group as a parameter of priority in vaccination against the coronavirus was the high morbidity and mortality in this group [9, 26]. This phenomenon occurs given the increased vulnerability to infectious diseases with unfavourable prognoses, added to associated comorbidities [27–29], as evidenced in April 2021, when 69.3% of SARS deaths were in individuals over 60 years of age, of which 61% had at least one comorbidity. Heart disease and diabetes were the most frequent conditions, and most of those who died and presented some comorbidities were 60 years or older [30].

In Brazil, by the end of April 2021, the first dose of CoronaVac or AstraZeneca vaccines had been administered to 24,768,325 individuals over 60 years, corresponding to 82.02% of the population estimate of the national COVID-19 vaccination campaign for this group [9]. In comparison with the United States, by 10 April 2021, 79.1% of the population above 65 years had initiated their vaccination [31]. This significant percentage of vaccinated elderly, with possible hospitalisation prevention of up to 94% with AstraZeneca [32] and 87.5% for CoronaVac [33], may be a relevant cause of the demographic shift in the profile of those affected by COVID-19 in 2021, with young and middle-aged adults representing a larger share of hospitalised cases and deaths. As shown in the Fiocruz epidemiological bulletin for weeks 16–17 of that year [34], the mean age of hospitalisation reduced from 62.7 to 57.1 and the mean age of deaths from 71.5 to 63.7 between epidemiological weeks 1 and 16.

Vaccination began in the Southeast region with the CoronaVac vaccine produced by Sinovac in partnership with the Butantan Institute. Such local production may explain why the Southeast region accounted for most of the vaccination by the elderly group (46.1%) and health workers (45.3%). The temporal distribution of vaccine doses followed the expected model, with clusters of high incidences of vaccination by EP occurring in epidemiological weeks 8 to 12, followed by high clusters of indigenous and quilombola group and health workers group, order of priority established by the national Ministry of Health [8]. Regarding the type of vaccine applied, the increase in high vaccination clusters was also due to the arrival of AstraZeneca/Oxford in Brazil on 21 March 2021 [35].

A decrease in the VR of CoronaVac was observed from week 14 to the last week analysed. It is important to point out that from May on, 72% of the vaccines distributed to the states were AstraZeneca type due to the decrease in the production of CoronaVac as a result of lack of inputs. This scarcity could be related to the diplomatic conflicts between the Brazilian government and the government of China, the leading supplier of Active Pharmaceutical Ingredient (API) to produce the CoronaVac vaccine [36]. The supply shortage can justify the predominance of high clusters of AstraZeneca vaccines over Coronavac across all groups starting on week 10, as evidenced in Fig. 1.

A possible cause pointed to the reduction in the vaccination rate for the EP was the inclusion of the group of comorbidities. This inclusion supposedly deviated the limited vaccine supplies to meet this new demand and could have caused the loss of vaccines since this group required medical statements before scheduling, which discouraged vaccination [37]. However, this argument becomes erroneous since, regardless of the recommended age range, chronic respiratory and cardiovascular diseases are presented as a high-risk factor for mortality by COVID-19 [8, 38]. Therefore, not including these groups in exchange for greater logistical ease would ignore the main objective of vaccination, which is to prevent severe cases and deaths.

Regarding the second dose, the elderly group in the Southeast region presented outstanding clusters of low rates for both vaccines, which can be related to the high number of people overdue for their second dose of vaccination in those states [39]. Furthermore, the difference in time between the first and second dose clusters, comparing the two vaccines, can be justified by the difference between the vaccination interval, which is 21 days between doses for CoronaVac and three months for AstraZeneca. Therefore, considering AstraZeneca's inter-dose interval, the clusters of the second vaccination dose were affected, given the time frame adopted in the current study.

Concerning spatial analysis, for the elderly group vaccinated with CoronaVac, spatial clusters of high VR were detected in the country, emphasising Ceará, Rio Grande do Norte and Paraíba, the eastern of Minas Gerais, and the northeastern portion of Rio Grande do Sul. An important fact is that Ceará has prioritised the elderly over 75 years as a first phase vaccination goal by EP before including the group under 60 years and comorbidities [9]. With AstraZeneca, it was observed that the countryside of Pernambuco, Alagoas and Sergipe, the eastern border between Minas Gerais and São Paulo, had the highest concentration of high VR among the clusters found. To the same priority group, the second dose of AstraZeneca showed a cluster of high VR points only in the state of São Paulo. This phenomenon may be supported by the fact that AstraZeneca application began earlier and on a larger scale after the lack of CoronaVac.

About ten weeks after the period analysed in the present study, health scenarios indicated a return in the relative increase of deaths and hospitalisations among the elderly. According to the Fiocruz Bulletin of the epidemiological week (EW) 31–32, this group returned to present an average near the 70 years in deaths. Furthermore, the proportion of hospitalised cases among the elderly, at 27.1% in EW 23, grew to 43.6% [39]. This change is due to the shift generated by the achievement of broader vaccination coverage for the elderly in lower age groups, together with a possible reduction in the immunising power over time, associated with a reduction in the potential of the immune system with advancing age and the emergence of more transmissible, and new variants, such as the delta variant.

By the end of May 2021, 11,965,603 healthcare workers had received at least one dose of CoronaVac or AstraZeneca. This group's pattern of vaccination distribution followed the expected demographic density arrangements in most of the country, with the Southeast leading the vaccinated contingent due to the higher number of healthcare workers (Table 1). The analyses of spatial clusters also detected this trend, especially for the state of São Paulo, where the Butantan Institute is located. This institute has successfully brought international partnerships for the development of tests and application of vaccines [41], highlighting the performance of CoronaVac, which generated, for both doses, spatial clusters of high vaccination rates in that state.

Right behind the Southeast, the Northeast region accounted for the second-highest number of vaccinated health workers in the country. However, for the North and South regions, the VRs observed with AstraZeneca were not higher than those expected for many HR. For its second dose, it was observed that healthcare workers remained leading in clusters of low VR in Brazil. This event was due, among other reasons, to the period covered by the study and to political and institutional disagreements [42–44], which ended up putting scientific negationism and ideological aspects ahead of vaccination objectives.

In addition, due to its continental dimensions and with multiple points of tension outside the political arena, Brazil needed to seek very efficient and coordinated logistical solutions for distribution and storage. In this sense, small failures accumulated throughout the process ended up causing delays and losses during the stages of immunisation, making the country quickly susceptible to the enormous human and material impacts of the pandemic during almost the entire year of 2020 [42–44]. This occurrence can be verified in the vaccination proportional to the population ranking [45], in which Brazil ranked 78th at the beginning of June 2021. Moreover, factors such as failures of shared management between the public administration levels; disparity and conflict between the healthcare capacity offered by public and private health systems; and actions that contributed to the country's political and economic instability, hindering more accurate and anticipated decision-making [42–44].

Vaccination coverage among healthcare workers offers a simultaneous benefit to patients and workers themselves. Studies have shown that the risk of healthcare workers becoming infected with the novel coronavirus was high but reduced considerably in this population after immunisation [46]. However, a study identified disparities between vaccination rates among healthcare groups, with physicians more vaccinated, followed by nurses and technical assistants [47].

Worldwide, priority groups of older people and healthcare workers were the first to benefit, followed by those with severe comorbidities [48, 49]. However, during the early stages of vaccination, these priority groups encountered several difficulties arising from the challenges of concatenating a limited amount of vaccines for many people with closely related needs [48–50]. The VR observed among healthcare workers in Brazil in the first months of immunisation was negatively distinguished from other large-sized countries, for example, with the rate found in the USA [51]. This event was related to the Brazilian government's difficulty in articulating the purchase of large quantities of vaccines in the first opportunities, which ended up not only delaying the vaccination for the priority groups but also overloading the vaccination sites, which had only CoronaVac available, therefore not meeting the massive demand [42–44].

The NASEM (National Academies of Sciences, Engineering, and Medicine) and the WHO have recommended that policymakers prioritise racial and ethnic minorities for vaccination against COVID-19 [52]. An analysis of indigenous populations in 23 countries found worse health indicators outcomes in this group: life expectancy at birth, infant mortality rate, maternal mortality, low birth weight, infant malnutrition, obesity, and others [53]. In Brazil, infectious and parasitic diseases are among the most responsible for indigenous deaths, especially compared to the rest of the national population. A recent example is the H1N1 pandemic in 2009, when 1,567 deaths among indigenous peoples were recorded in Brazil, 4.5 times more affected than the general population [13].

In the current pandemic, the greater vulnerabilisation of certain racial and ethnic groups is a reality worldwide and nationally. For example, a study published in late 2020, which analysed 29,008 cases and 532 deaths of indigenous people in Brazil from COVID-19, found national incidence and mortality rates of 3,546.4 cases and 65.0 deaths per 100,000 inhabitants, respectively, and a case fatality rate of 1.8% in the group [14, 54]. Factors that explain the vulnerability produced to this group in the current pandemic in the country are: communal lifestyle; constant invasions of indigenous lands; missionary actions; absence of a protection policy by the federal government; lack of an adequate drinking water supply system; poor access to hygiene products and alcohol gel; limited access to health care, especially those of high complexity [13]; and reduced investments of the *Mais Médicos* program, which has resulted in the dismissal of more than 8.000 primary care physicians in 2019, mainly affecting indigenous communities in remote areas [54].

The present study found clusters of high vaccination rates with CoronaVac during the first seven epidemiological weeks and low vaccination rates in the following weeks. With AstraZeneca, high vaccination clusters occurred after the tenth week. This temporal pattern followed the expected trend for priority groups included in the first phases of vaccination in most state and municipal plans. Moreover, it was very similar to the temporal pattern found for healthcare workers. Among the groups analysed in this study, the indigenous and quilombola populations had the lowest vaccination rates, with a national rate of 0.62/100.

The percentages of complete vaccination schedules were also the lowest when comparing indigenous and quilombola to the other groups studied, not exceeding 2% in any of the five regions during the weeks studied. In the North region, for example, where 97.3% of demarcated indigenous lands in the country are concentrated [55], the vaccination rate for the group was 0.18/100 inhabitants, with 0.38% of individuals with a complete scheme. Some difficulties faced during the period studied for the vaccination of these ethnic and racial groups may explain such results: deficient national immunisation plan, highlighted by inconsistencies regarding the definition of priority groups; geographic isolation; problems with local management and vaccine hesitation.

Regarding the indigenous population, the Special Secretariat for Indigenous Health (SESAI) is responsible for the immunisation actions executed by 34 Special Indigenous Health Districts (DSEI) [55]. For the establishment of the National Plan for the Operationalisation of vaccination against COVID-19, the estimation of the indigenous population was based on data from the Department of Indigenous Health (DESAI), including those over 18 years of age served by the national public health system, living on demarcated lands.

According to the Plan, it was recommended that the vaccination should be carried out in agreement with the DSEI responsible for each municipality with which the indigenous lands are associated, including this population in the vaccination first phase [9]. There are 310 indigenous lands in stalled demarcation processes. Consequently, in this first stage, 379.5 thousand people, or 42.8% of the indigenous population, were left out of the first stage of the immunisation against coronavirus [55]. On 16 March 2021, the Federal Supreme Court (STF) accepted the appeal of the Association of the Indigenous Peoples of Brazil (APIB), determining that even "unallocated" indigenous people should be included in the General Plan of Confrontation of COVID-19 for Indigenous Peoples [56], leading to an increase in the vaccination following the decision.

For the quilombola population, data from the 2010 National Census were applied regarding individuals above 18 years of age, maintaining the recommendations for creating strategies at the municipal level [9]. The quilombola population also faces problems regarding the certification of their territories. The titling of lands belonging to the remaining quilombola peoples/communities is carried out by the National Institute of Colonisation and Agrarian Reform (Incra), and less than 7% of them are regularised. The populations of lands whose possessions are not regularised found, in the pandemic, the problem of access to vaccination, testing and medical assistance [57]. Moreover, in the 23 national states that have made available their vaccination plans, only 12 have included the quilombola population among the priority groups. The majority did not inform the phase in which the vaccine should be applied in this group [13].

Regarding both groups, geographic isolation imposes itself as another difficulty faced. The Associated Press, which accompanied the transport of vaccines by health workers to indigenous and quilombola communities, reported problems regarding the delivery of vaccines in locations with access only possible by boat, requiring several days of travel, highlighting the difficulty of maintaining the refrigeration of vaccines during transport [58]. For example, in the *quilombo Kalunga do Mimoso*, in the state of Tocantins, the community had to gather to purchase a refrigerator to store the vaccines since the municipality provided the doses but not the refrigeration equipment. In summary, the vaccination of ethnic priority groups in the country is hindered by local public administrators' lack of political will [59].

According to the last national census (2010), the regions with the largest indigenous populations are the North, with 342,836 people and the Northeast, with 232,739 [60]. Concerning the quilombos, the states of Bahia (NE), Maranhão (N) and Minas Gerais (SE) concentrate more significant numbers; also highlighted are other states of the Northeast region, and the state of Rio Grande do Sul [61]. However, despite presenting higher concentrations of demarcated lands and people declared or considered indigenous, the North region did not present clusters of high or low vaccination rates. The absence of high vaccination clusters can be explained by the problems previously mentioned, especially in the Legal Amazon region.

Despite the significant indigenous and quilombola populations contingent, which could translate into lower vaccination rates due to the greater need for vaccines, the Northeast region presented high vaccination clusters for both vaccines. With the pandemic of COVID-19, the matter of vaccine hesitancy returned strongly to the scientific community's interest, which moved new efforts to investigate the rates of vaccine intention [62]. Research involving healthcare workers showed that the rates of intention to be vaccinated among professionals varied throughout the pandemic between 27.7 and 77.3% [63]. Among the main issues involved in vaccine hesitancy are insecurity about its efficacy/quality and the political and organisational dissonance between governmental sectors, health entities and supranational bodies [63, 64]. This social behaviour may have reduced VR among health workers in some country areas.

For the elderly, a relevant issue directly related to the low demand for the second dose is the wide dissemination of fake news and the movement of anti-vaccine groups. Despite studies that prove the vaccine's effectiveness, vaccine hesitation is still frequent, and anti-vaccine movements can find even greater ground among the elderly group since they also represent the age group with less access to education and high rates of functional illiteracy [64, 65].

In certain indigenous localities, vaccine hesitancy and resistance are of particular concern, especially in the states of the legal Amazon. According to SESAI, 70% of the indigenous people living in the Rio Tapajós basin villages (state of Pará) were doubtful or had refused to take the COVID-19 vaccine. In the region, 6,420 doses of the vaccine were made available by 25 March 2021. However, only 30.3% were applied, as 35.2% of the indigenous people refused to take it, and another 32% stated they were hesitant about accepting the immunisation [66]. The dissemination of fake news by evangelical religious groups has been pointed out as one of the main factors responsible for such hesitation, especially in the states of the legal Amazon, where these groups have spread in the last ten years. An analysis with community leaders, researchers, indigenous people, and non-governmental organisations showed that where there is a more significant presence of pastors opposed to vaccination, VRs were lower [67].

While collecting data for the study, some limitations were identified. The database used had some discrepancies in the notification, such as the non-matching of the number of doses applied and people associated with risk groups, making it difficult to interpret some vaccination behaviours, especially for the population over 80 years old in the South region. In addition, another limitation was the absence of demographic data for the year 2021, which was replaced by 2020 values. Likewise, the demographic data for indigenous and quilombola people for 2019 were used, the most up-to-date available. From the study period perspective, while this work was able to broadly overview the behaviour between doses of CoronaVac, the evaluation of the dynamics between doses of AstraZeneca was hampered, given that at the end of the 21st epidemiological week of 2021, this vaccine had not yet reached the second dose's peak of administration.

## Conclusions

The present study found clusters of high vaccination rates with the CoronaVac vaccine during the first half of the epidemiological weeks analysed, followed by low vaccination rates in the remaining weeks. For the AstraZeneca vaccine, high vaccination clusters occurred after the tenth week. This temporal pattern followed the expected trend for the order of priority population groups established by the national Ministry of Health, included in the first phases of vaccination in most state and municipal plans.

The Southeast region accounted for most of the vaccination by elderly group and health workers in the period studied. Regarding the second dose for the EP, this region presented multiple clusters of low rates for both vaccines. For the HW group, the pattern of vaccination distribution followed the expected demographic density arrangements in most of the country, with the Southeast leading the vaccinated, followed by the Northeast region. Spatial clusters also detected this trend, especially in the state of São Paulo, where the Butantan Institute is located and has successfully brought international partnerships for the national vaccination.

Among the groups analysed in this study, the indigenous and quilombola populations had the lowest vaccination rates in all five regions of Brazil, including the percentages of complete vaccination schedules. The study results highlight the process of vulnerabilisation of certain racial and ethnic groups, intensified by the pandemic.

Considering the continental dimensions of the country and the multiple points of tension outside the political arena, Brazil needed a very efficient and coordinated logistical solution for distribution and storage. The analysis by spatial and temporal conglomerates allowed the weaknesses detection of the national vaccination campaign against COVID-19, from the determination of who were the priority groups initially immunised, how they were distributed in the national territory, and how the temporal dynamics of this immunisation occurred. The verified scenario showed accumulated failures throughout the process, causing delays and losses during the immunisation process, making Brazil quickly susceptible to the massive and multiple impacts of the COVID-19 pandemic.

## Data Availability

The datasets analysed during the current study are available in the following Brazilian public repositories. OpenDataSUS, https://opendatasus.saude.gov.br/dataset/covid-19-vacinacao/resource/ef3bd0b8-b605-474b-9ae5-c97390c197a8; DATASUS-Tabnet-CNES-Estabelecimentos, http://tabnet.datasus.gov.br/cgi/deftohtm.exe?cnes/cnv/estabbr.def; DATASUS-Tabnet-CNES-Recursos Humanos, http://tabnet.datasus.gov.br/cgi/tabcgi.exe?cnes/cnv/prid02br.def; IBGE-Base de Informações sobre os Povos Indígenas e Quilombolas 2019, https://www.ibge.gov.br/geociencias/organizacao-do-territorio/tipologias-do-territorio/27480-base-de-informacoes-sobre-os-povos-indigenas-e-quilombolas.html?=&t=downloads; IBGE-Cadastro de Localidades Indígenas em 2019, https://dadosgeociencias.ibge.gov.br/portal/apps/opsdashboard/index.html#/2a92001820fd4f3894cfbd41c5e91a02.

## Abbreviations

APIB: Association of the Indigenous Peoples of Brazil
CW: Central-West region
DESAI: Department of Indigenous Health
DSEI: Special Indigenous Health Districts
EP: Elderly population
EW: Epidemiological week
HR: Health Regions
HW: Healthcare workers/professionals
I/Q: Indigenous and quilombola populations
IBGE: Brazilian Institute of Geography and Statistics
Incra: National Institute of Colonisation and Agrarian Reform
MH: Ministry of Health
N: North region
NE: Northeast region
PNI: National Vaccination Programme
RR: Relative risk
S: South region
SARS: Severe acute respiratory syndrome
SARS-CoV-2: Severe acute respiratory syndrome coronavirus 2
SE: Southeast region
SESAI: Special Secretariat for Indigenous Health
STF: Federal Supreme Court
VR: Vaccination rates
WHO: World Health Organization
